# A metapopulation model for highly pathogenic avian influenza: implications for compartmentalization as a control measure

**DOI:** 10.1017/S0950268813002963

**Published:** 2013-12-05

**Authors:** S. NICKBAKHSH, L. MATTHEWS, S. W. J. REID, R. R. KAO

**Affiliations:** 1 Institute of Biodiversity, Animal Health and Comparative Medicine, University of Glasgow, Bearsden Road, Scotland, UK; 2 Royal Veterinary College, University of London, North Mymms, Hatfield, Hertfordshire, UK

**Keywords:** Avian influenza, mathematical modelling, notifiable infectious diseases, Susceptible-Infected-Removed (SIR) model, veterinary epidemiology

## Abstract

Although the compartmentalization of poultry industry components has substantial economic implications, and is therefore a concept with huge significance to poultry industries worldwide, the current requirements for compartment status are generic to all OIE member countries. We examined the consequences for potential outbreaks of highly pathogenic avian influenza in the British poultry industry using a metapopulation modelling framework. This framework was used to assess the effectiveness of compartmentalization relative to zoning control, utilizing empirical data to inform the structure of potential epidemiological contacts within the British poultry industry via network links and spatial proximity. Conditions were identified where, despite the efficient isolation of poultry compartments through the removal of network-mediated links, spatially mediated airborne spread enabled spillover of infection with nearby premises making compartmentalization a more ‘risky’ option than zoning control. However, when zoning control did not effectively inhibit long-distance network links, compartmentalization became a relatively more effective control measure than zoning. With better knowledge of likely distance ranges for airborne spread, our approach could help define an appropriate minimum inter-farm distance to provide more specific guidelines for compartmentalization in Great Britain.

## INTRODUCTION

Within the context of controlling notifiable livestock disease epidemics, the World Organization for Animal Health (OIE) stipulates that ‘establishing and maintaining a disease-free status throughout the country should be the final goal for OIE members’ (chapter 4·3, OIE Terrestrial Animal Health Code [1]). However, these guidelines also recognize the difficulty in ‘establishing and maintaining a disease-free status for an entire territory’ and therefore promote the benefits of establishing and maintaining *subpopulations* of animals with a distinct health status. These subpopulations are usually defined through *zoning* (or *regionalization*), where geographical boundaries separate parts of a territory (or country). An example is the implementation of control zones used to enforce movement restrictions and enhanced biosecurity measures during outbreaks of notifiable disease [2].

The relatively recent concept of *compartmentalization* extends this idea by considering pathways other than geographical proximity that may jeopardize the biosecurity of a compartment. The OIE guidelines stipulate that ‘the definition of *compartment* may revolve around disease-specific epidemiological factors, animal production systems, biosecurity practices, infrastructural factors and surveillance’ (chapter 4·4, OIE Terrestrial Animal Health Code [3, 4]). These, generally speaking, non-spatial factors include the movements of animals, equipment, other fomites, feed and people. Biosecurity considerations will also include spatial proximity to adjacent animal populations; in this respect *compartmentalization* is comparable to *zoning* control.

The major benefit of compartmentalization is the potential continuation of exports from compartments during an outbreak (upon agreement with the trade partners) and it is this economic aspect that is driving the growing interest in European Union Member States, and their poultry and swine production industries, in adopting this concept [5]. Compartmentalization is also a means of outbreak prevention by minimizing spatial- and fomite-mediated mechanisms of spread and can facilitate the eventual elimination of disease by concentrating personnel and resources in areas where elimination is most likely to succeed [6].

Compartmentalization is not conceptually new given that, historically, disease control programmes have been based on the concept of disease-free herds or flocks [3]. However, compartmentalization has not yet been formally appraised, nor has it been applied, so currently there are no examples of success and any potential associated risks are difficult to determine. Appraisal is made all the more difficult because the requirements for compartment status are not specific, yet it is recognized that the implementation of both compartmentalization and zoning will be influenced by factors specific to the pathogen, industry and country in question [6].

Of particular concern for governments worldwide in relation to commercial poultry production is the global spread of avian influenza, particularly in its highly pathogenic form (HPAI). The OIE has produced guidelines on the practical implementation of compartmentalization for avian influenza and Newcastle disease (which is another important notifiable disease of poultry) [7], with consideration of the specific risks of entry and spread of avian influenza. A better understanding of the potential epidemiological links, both through spatial proximity and through movements of people, vehicles and equipment [8–10], could help provide a more specific definition of compartmentalization for the British commercial poultry industry.

Given the current lack of appraisal of the application of compartmentalization, and the potential need to refine the definition under different disease, industry and country-specific circumstances, we aim to provide an example modelling framework developed to investigate the risks of infection leakage from compartments within the British commercial poultry industry.

We focus our analyses on integrated poultry companies (premises with common ownership) as they are suitable candidates for compartmentalization due to the vertical integration of their business [6]. We explore a range of outbreak scenarios, characterized by their basic reproduction number (*R*_0_), in order to provide a quantified measure of risk under compartmentalization in comparison to that under zoning control.

## METAPOPULATION FRAMEWORK

### Network characterization

The Poultry Network Database (PND) was collated in 2006 by the Veterinary Laboratories Agency (now known as Animal Health and Veterinary Laboratories Agency; AHVLA) and describes the poultry premises (*n* = 4067), which are the clients of major slaughterhouses (SH; *n* = 96) and catching companies (CC; *n* = 102) within the Great Britain (GB) commercial poultry industry [8, 11]. These SH–client and CC–client associations were assumed to represent a potential epidemiological link representing the risk for pathogen spread via fomites during on-farm catching-team visits, particularly during flock-thinning which is carried out mid-production cycle (i.e. risks via personnel, clothing, equipment, forklift trucks, slaughterhouse vehicles). We note the activity of catching teams is recognized to be relevant to the potential risk of avian influenza spread within the GB poultry industry [8, 12].

We define a poultry company as a group of multiple individual premises under common ownership which make up the individual components of a compartment; these individual premises we denote multi-site (MS) premises. Overall, MS premises status was highly correlated with membership of an integrated company (Pearson's product-moment correlation coefficient = 0·92, 95% CI 0·91–0·93, *P* < 0·0001). We generated a bipartite network where premises and SHs (or CCs) represented nodes and the SH–client (or CC–client) associations informed the links between the nodes. This network characterization was then used to generate an unweighted and undirected unipartite network where a single set of nodes represented poultry companies and links represented shared SH or CC associations (see [13]). If at least one premises from each of two companies shared a SH (or CC), the respective companies were deemed to be associated.

**Fig. 1:**
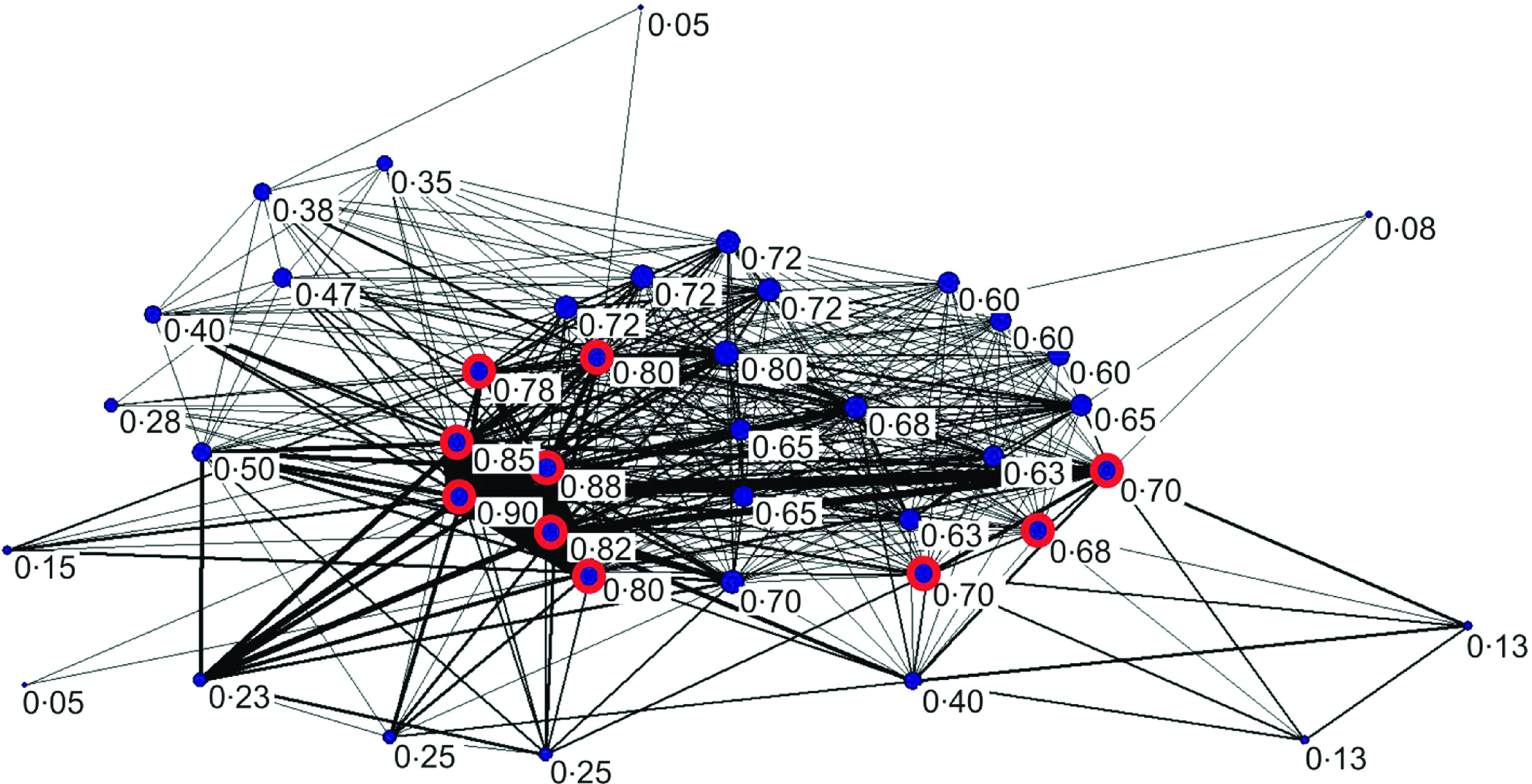
[*colour online*]. Unipartite representation of the poultry company network of associations via slaughterhouses and catching companies; *n* = 41 poultry companies (excluding four that were unconnected to the giant component). Node labels are the normalized (by largest possible value) degree centralities with relative magnitudes represented by node size. Nodes circled in red represent companies which also had a relatively high betweenness centrality. The line widths represent the number of slaughterhouses and/or catching companies that formed the associations.

Figure 1 shows that the company-level GB poultry network was highly connected forming one giant component (consisting of 91% of premises in the PND). The network giant component represents the largest of all possible components [15]. Each individual node within a giant component can reach all other nodes via at least one path, providing an estimate for the maximum possible epidemic size for networks representing disease transmission [14, 15]. This high connectivity has important implications for the nature of epidemiological risks that should be considered in the definition of compartmentalization for GB.

The topological properties of a network can be quantified by a number of measures. The most widely used measures the centrality, or importance, of the network node. The degree centrality, in its most common form, measures the total number of nodes directly adjacent to each node [16]. Other measures consider network paths; these quantify the routes across the network from one node to another via each link, e.g. the betweenness centrality quantifies the number of shortest paths between pairs of nodes [17].

In this study we used degree and betweenness as measures of network centrality computed using Pajek v. 2.04 software [18]. The network giant component could be divided into two main subgroups: (i) a *core group* of companies with relatively high degree centrality, some of which also had relatively high betweenness (see inner nodes circled in red with degree centrality, normalized by the largest value, >0·6, Fig. 1), and (ii) a *periphery* group of companies with both relatively low degree and betweenness centralities (see outer nodes with degree, normalized by the largest value, <0·6, Fig. 1). We used these network links between companies to inform a metapopulation model of HPAI transmission with connections between two populations; one population represents a company/compartment within the core network group and the other represents a company/compartment within the periphery network group.

### Population structure

We aimed to capture the potential for spread between MS premises (that are under common ownership, i.e. a compartment) and other premises at risk of infection. As single-site (SS) premises tended to be spatially clustered around MS premises (80–90% of premises within the proximity of MS were SS premises across distances of 1–10 km), we captured the potential for transmission between MS and SS premises both within and between the two compartment populations. More specifically, each population comprised of the MS premises of a single company and nearby SS premises. Hereafter *ρ* = 1 denotes a company within the network *core* group and *ρ* = 2 denotes a company within the network *periphery* group.

Interrogation of the PND showed that the mean number of premises for companies within the core and periphery groups were 70 and 10, respectively, reflecting the typical size of a poultry company within the respective network groups. These data regarding company size informed the number of MS premises within population 1 (*n*_1,MS_ = 70) and population 2 (*n*_2,MS_ = 10). The mean number of SS premises (*n*_SS_) in the proximity of MS premises (*n*_MS_) was determined for each population across varying distance radii, corresponding with assumptions considered for airborne transmission of HPAI. Results are shown for 1 km radii (corresponding with airborne transmission up to 1 km) where *n*_SS_<*n*_MS_ (*n*_1,SS_ = 35 for population 1 and *n*_2,SS_ = 5 for population 2).

### Transmission mechanisms

**Fig. 2.**
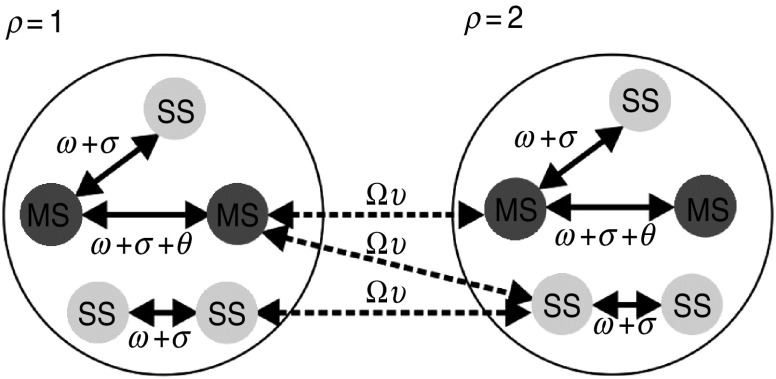
Schematic of the metapopulation model. The compartment populations represent the multi-site (MS) premises associated with a company from the network *core* (*ρ* = 1) and the network *periphery* (*ρ* = 2), and the single-site (SS) premises within proximity. Solid arrows = within-population links; dashed arrows = between-population links; Ω = between-population network link weights; *υ* _=_ between-population interaction strength; *ω* _=_ within-population network link weights; *θ* = company-related network link weights and *σ* = within-population spatial link weights. See Table 1 for full parameter details. Note that this schematic does not reflect the actual relative numbers of MS and SS premises assumed in these analyses.

Figure 2 provides a schematic representation of the model structure. Two mechanisms at the farm-level that could potentially allow for HPAI introduction onto a farm are: (i) onto-farm movements related to the catching and transportation of birds to the slaughterhouse (i.e. both the catching team and slaughterhouse vehicle components of these on-farm visits), referred to hereafter as ‘network-mediated links’, and (ii) airborne or environmental spread, referred to hereafter as ‘spatially mediated links’.

Network-mediated links between farms occurring through slaughterhouses and catching companies were inferred from the PND (*n* = 3044 farms) as described previously [see 11]. Spatially mediated links between farms through proximity were inferred from the geographical locations provided by the PND (*n* = 3153) and the Great Britain Poultry Register (*n* = 1991). The threshold for spatially mediated links was varied in a sensitivity analysis due to a poor understanding of the likely distance for airborne spread in GB. For most farms, the between-farm distances were relatively large (>150 km), although 67% of farms had at least one neighbour within 3 km suggesting there is potential for airborne transmission between these premises. Based on the experience of The Netherlands, which was afflicted by a major outbreak of HPAI in 2003, evidence of meaningful transmission beyond 10 km is limited [19, 20], therefore we considered distances from 1 km to 10 km and present results for 1 km.

Within a population, MS premises could transmit infection to other MS premises, as well as to SS premises via both network and spatial links. MS premises could also transmit infection within their company, representing transmission via shared staff and equipment [21]. Similarly, SS premises could transmit to other SS premises as well as to MS premises within the same population via both network and spatial links. Between-population transmission events were assumed to only occur via network links, reflecting the observation that MS premises were less likely to be within close proximity of the MS premises of another company, thereby limiting the potential for airborne spread.

### Informing contact heterogeneity

Pairwise matrices summarizing the links between premises via network-mediated and spatially mediated mechanisms of contact were used to determine the proportions of contact, both within and between populations, in the metapopulation model. Specifically, *σ*_*jk*_ is the proportion of all spatially mediated links from premises of type *k* to premises of type *j, ω*_*jk*_ is the proportion of all network-mediated links *within a compartment population* from premises of type *k* to premises of type *j* and Ω_*jk*_ is the proportion of all network-mediated links *between compartment populations* from premises of type *k* to premises of type *j* (where *j, k* take values MS premises or SS premises).

Appendix A in the Supplementary online material provides further details of how these link-type probabilities were scaled to the distribution of MS premises and SS premises according to the more demographically representative Great Britain Poultry Register (see [11] for details regarding demographic biases in the PND). These link-type probabilities were used to apply a weighting to the transmission rates in the Susceptible-Infected-Removed (SIR) model (described below). All network and spatial links were assumed to be active at any given time; in the case of the network links, this is equivalent to assuming that visits are relatively frequent compared to infectious periods, and infectious connections between associated premises are random. As this approximates many of the properties of homogeneous mixing within groups [22], the model therefore provides an insight into outbreak risk under a scenario of maximal connectivity between poultry premises.

**Fig. 3:**
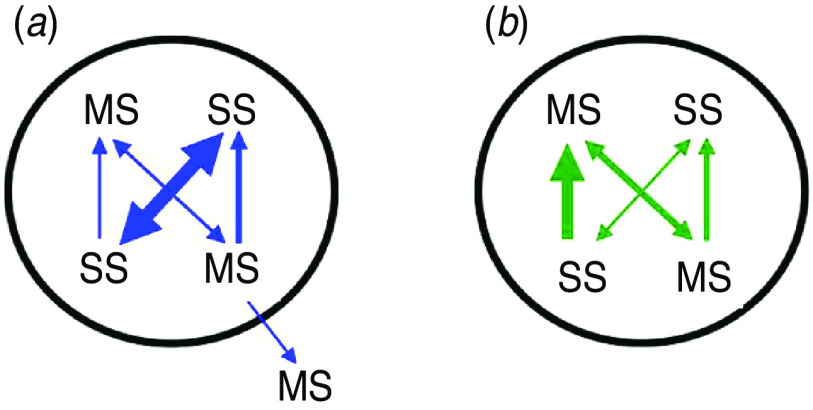
[*colour online*]. Schematic of the relative link-type weights. (*a*) network-mediated links, (*b*) spatially mediated links (representing proximities ranging 1–10 km). The premises types are represented by multi-site (MS) and single-site (SS) premises. Relative arrow sizes represent the link-type weights for the corresponding transmission mechanism (see Table 2).

Link-type probabilities are later used to weight the transmission rates informing heterogeneous mixing within and between populations in the metapopulation model. Figure 3 shows all possible link-types for a single population, comparing the two transmission mechanisms. Network-mediated links were predominantly between SS premises, whereas for spatially mediated links this was the least probable and links between MS premises and SS premises predominated. For MS premises, spatially mediated links to the MS premises of another population (i.e. between-company) was relatively more probable than with MS premises of the same company for distances up to 3 km.

### The SIR models

Disease transmission over the metapopulation model was implemented using a deterministic SIR compartmental framework. The total number of premises within each infection state per premises type and population were tracked throughout the duration of an outbreak. The transition to compartment ‘R’ represented disease detection and subsequent total flock depopulation following the implementation of control measures which was assumed to occur at rate *γ* (days^−1^). Once premises reached this status, they posed no further transmission risk to other premises through either network or spatial mechanisms. We considered a range of values for *γ* (0·33, 0·25, 0·17 day^−1^), given by the reciprocal of anticipated farm-level infectious periods, ranging 3–6 days consistent with previous models of the between-farm spread of avian influenza in GB [23, 24]. It was assumed that repopulation of premises occurred outside of the time-frame of an outbreak.

The baseline model for the interaction between population 1 (network *core* group) and population 2 (network *periphery* group) is described by the following set of ordinary differential equations:

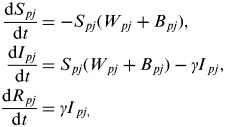
where *ρ*={1, 2} for population 1 (network *core*) and population 2 (network *periphery*) and *j*={MS, SS} for multi-site and single-site premises types and *W*_*ρj*_ and *B*_*ρj*_ represent the within- and between-population forces of infection acting upon premises type *j* within population *ρ*.

The force of infection acting on *j* = MS premises within *ρ* = 1 (network core group) is further deconstructed as follows:

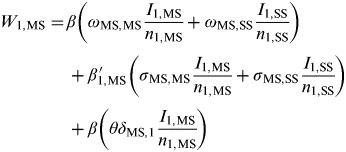


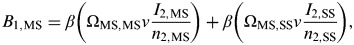
where *β* and *β′*_1,MS_ are density-independent and density-dependent transmission rates (days^−1^), respectively, *σ*_MS*,k*_ and *ω*_MS*,k*_ weight the rate of transmission from premises type *k*={MS, SS} to MS premises through spatial proximity and network links respectively, *θ* weights the rate of transmission between MS premises of the same company, *δ*_MS,1_ is the Kronecker delta function, where *δ*_*jk*_ = 1 if *j* = MS premises or zero otherwise, Ω_MS*,k*_ weights the rate of transmission between populations and *υ* is a uniform weighting applied to Ω_MS*,k*_ in order to vary the relative strength of transmission between populations (i.e. the interaction, or coupling, strength). These equations can equivalently represent the force of infection acting on SS premises by substituting *j* = SS premises and for population 2 by substituting *ρ* = 2.

The density-dependent transmission rate *β′*_*ρj*_ is given by 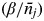
*n*_*ρj*_ where the density-independent transmission rate *β* (days^−1^) is now proportional to the number of premises of type *j* within population *ρ* (*n*_*ρj*_) and is further scaled by an overall median number of *j* premises (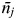) across all populations *ρ*. Given the lack of experience of large HPAI outbreaks in GB, and therefore the uncertainty in the pathogen's likely transmissibility, we explored a range of transmission rates (0·0063–0·93 day^−1^) that generated basic reproduction numbers (*R*_0_) ranging from <1 to 8 in accordance with the published literature investigating potential outbreaks of HPAI in GB, as well as the experiences of other countries [23–25]. Table 1 provides further details of the parameter values explored in these analyses.

**Table 1.** SIR metapopulation model notation, parameters and initial conditions

Parameter	Definition	Initial/default values/ranges	Reference/source
*n*_1,MS_	Total number of multi-site premises within population 1	70	PND
*n*_2,MS_	Total number of multi-site premises within population 2	10	PND
*n*_1,SS_	Total number of single-site premises within population 1	35	PND
*n*_2,SS_	Total number of single-site premises within population 2	5	PND
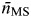	Median number of multi-site premises across all populations	15	PND
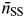	Median number of single-site premises across all populations	8	PND
*β*	Between-farm transmission rate (days^−1^) assuming density-independence	0·01–0·2, increments of 0·00255	[23–25]
*β*′	Between-farm transmission rate (days^−1^) assuming density-dependence*	0·0063–0·93, increments of 0·00255	–
*γ*	Rate at which infected farms were depopulated (days^−1^)	0·17, 0·25 or 0·33	[23, 24]
*ω*_*jk*_	Within-population proportion of network links from premises of type *k* to *j*, where *j,k*={MS, SS}	See Table 2	PND
*θ*	Within-population proportion of network links between MS premises only (i.e. within-company links)	1	Fixed†
*σ*_*jk*_	Within-population proportion of spatial links from premises of type *k* to *j*, where *j,k*={MS, SS}	See Table 2	PND
Ω_*jk*_	Between-population proportion of network links from premises of type *k* to *j*, where *j,k*={MS, SS}	See Table 2	PND
*ν*	Between-population interaction strength	0–10, increments of 0·1335	Fixed†
*δ*_*jk*_	Kronecker delta function used to ensure *θ* only applies to transmission within a company	0, if *j*=SS 1, if *j*=MS	−
*W*_*ρj*_	Within-population force of infection acting on premises *j* within population *ρ*	–	Simulated
*B*_*ρj*_	Between-population force of infection acting on premises *j* within population *ρ*	–	Simulated

PND, Poultry network database; MS, multi-site premises; SS, single-site premises.

* This parameter was informed by *β*, 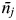 and *n*_*ρj*_.

† These parameters were fixed; an assessment of model sensitivity found these parameters to have little impact on the qualitative model results.

**Table 2.** Between-premises link-type probabilities via network and spatial mechanisms

Link type*	Network (*ω* and Ω)†	Network >10 km (*ω* and Ω)† ‡	Spatial (*σ*): 0–1 km†	Spatial (*σ*): 0–3 km†	Spatial (*σ*): 0–10 km†
*jk* = MS, MS§	0·366	0·389	0·088	0·043	0·020
*jk* = MS, MS¶	0·008	0·010	–∥	–∥	–∥
*jk* = MS, SS	0·431	0·450	0·373	0·362	0·247
*jk* = SS, MS	0·360	0·353	0·449	0·567	0·744
*jk* = SS, SS	0·879	0·842	0·0002	0·0002	0·0002

* *jk* represents all possible link-types between premises types *j* = {MS, SS} and *k* = {MS, SS}, where *jk* = MS, MS represents links between multi-site premises, *jk* = MS,SS and *jk* = SS,MS represents links between multi-site and single-site premises; *jk* = SS, SS represents links between single-site premises.

† See Appendix A (Supplementary material) for further details of how link-type probabilities were scaled to the expected distribution of multi-site and single-site premises according to the Great Britain Poultry Register. The scaled link-type probabilities were used to apply a weighting to the transmission rates in the SIR model.

‡ Used to inform network links under zoning in the control scenarios (see ‘*R*_0_ thresholds: compartmentalization’ section).

§ Within-compartment (or within company).

¶ Between compartment (or between companies).

∥ No spatial links occurred between compartment populations.

Although the likely mode of transmission of HPAI within GB is not well understood, our model was insensitive to the distance assumed for spatial transmission except under density-dependent transmission. We therefore present results for this scenario to demonstrate the potential for disease spread under maximal possible airborne spread for the distances considered. The overall potential for an epidemic of HPAI was represented by the between-farm *R*_0_, computed as the dominant eigenvalue of the Next Generation Matrix for the ODE model system. Further details of the calculation of *R*_0_ are provided in Appendix B (Supplementary online material).

### Defining control models

**Fig. 4.**
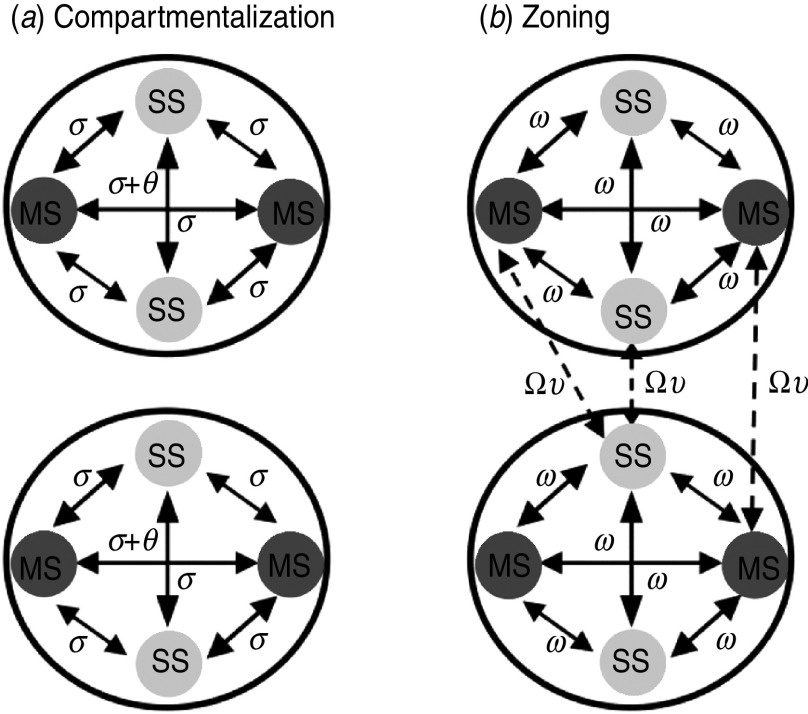
Schematic representation of zoning and compartmentalization control scenarios. (*a*) Under compartmentalization the following links were enabled: spatial and within-company links (*σ, θ*). (*b*) Under zoning control the following links were enabled: within-population network-mediated links (beyond 10 km) and between-population network links (*ω*, Ω*υ*).

The baseline model described above was adapted to assess the risk of an outbreak under compartmentalization. To provide practical insight, this risk was assessed through a relative comparison with a *zoning* control scenario. Figure 4 shows the outbreak interventions assumed. For compartmentalization, all network links within and between compartments were inhibited; however, the possibility remained of infection spillover between compartments and SS premises within proximity. Therefore we use a global value of *R*_0_ as a measure of when these spillovers may have a widespread impact. These conditions were motivated by the OIE guidelines for the epidemiological separation of compartments. The guideline for GB compartments specifies a minimum distance of 400 m to other premises [26]. Given that it is possible that airborne spread of HPAI could occur at distances beyond 400 m (based on the experience of other countries [19, 20, 27]), we explored the impact of allowing spatially mediated transmission to occur at distances >400 m.

In contrast, under zoning all spatial links, network-mediated links within 10 km and within-company links were inhibited thereby mitigating all transmission risks operating at relatively short distances. The purpose of zoning control is to enforce enhanced biosecurity and to limit movement of poultry between affected and non-affected areas (generally, in the case of HPAI, 3 km protection zones and 10 km surveillance zones are implemented which enforce different levels of restrictions [2]). The zoning scenario in these analyses corresponds to a specific situation where infection is assumed to have already spread beyond 10 km surveillance zones through the movements of fomites (i.e. people, vehicles, equipment), prior to the enforcement of control measures following identification of the index premises (see Table 2 for network link-type probabilities under zoning).

## *R*_0_ thresholds: baseline model

**Fig. 5.**
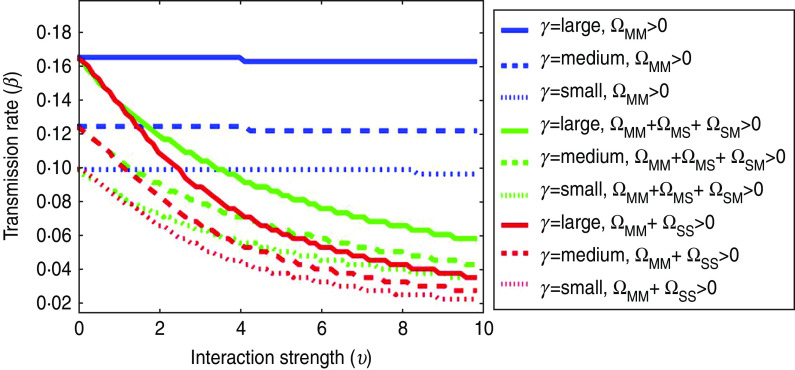
Sensitivity of *R*_0_ thresholds to farm-level parameters. *γ* = rate of premises depopulation (small = 0·17 day^−1^, medium = 0·25 day^−1^, large = 0·33 day^−1^; Ω_MM_ *=* links between multi-site premises, Ω_MS_ and Ω_SM_ = links between multi-site and single-site premises; Ω_SS =_ links between single-site premises. Link types not shown in the legend are switched-off (i.e. Ω = 0). This model scenario assumed density-dependent spatial transmission occurred within a distance radius of 1 km.

Figure 5 shows *R*_0_ = 1 thresholds for varying values of the transmission rate (*β*) and between-population interaction strength (*υ*). Parameter combinations above each line (i.e. where *R*_0_>1), indicate the potential for an outbreak, while parameter combinations below each line (i.e. where *R*_0_<1) indicate no outbreak potential. Each line corresponds to a different outbreak (informed by *β*) and control measure (informed by *υ*) scenario; the link types enabling a between-population interaction are represented by line colour, and the premises-level infectious periods (1/*γ*) are represented by line type.

The clustering of lines by colour shows that outbreak risk was most sensitive to the link types enabling between-population transmission and relatively less sensitive to the premises-level infectious period (1/*γ*). Transmission between poultry companies (i.e. Ω_MS_) alone was sufficient for *R*_0_ >1; additional transmission involving SS premises (i.e. Ω_MS_, Ω_SM_ or Ω_SS_) further enhanced this, an effect which increased with the interaction strength (*υ*). There was also little sensitivity to the assumed distances for spatially mediated links (see Table 2).

**Fig. 6:**
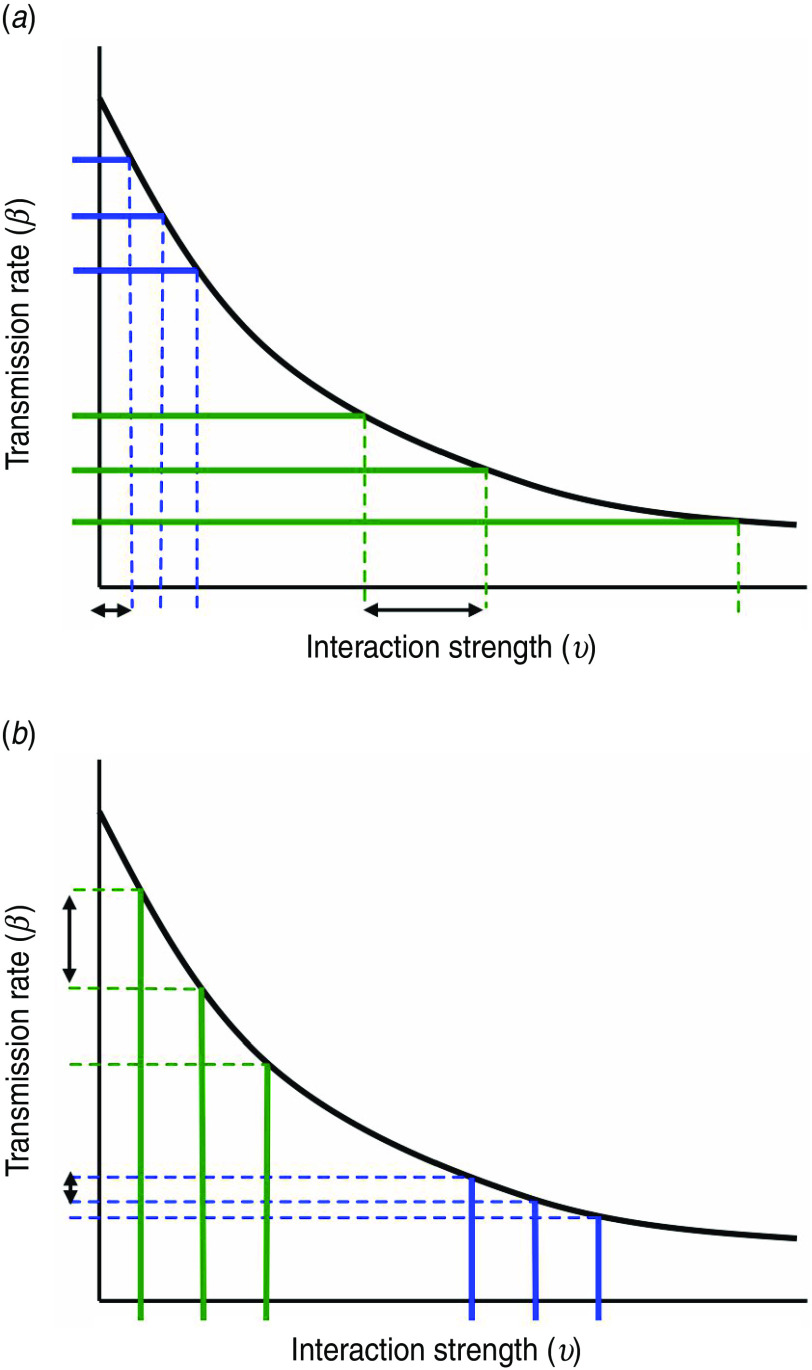
[*colour online*]. Schematic representation of how parameter trade-offs maintain outbreak potential. (*a*) When decreasing from high *β* (blue lines), only relatively small increases to *υ* maintains the *R*_0_ threshold, compared to decreases from small values of *β* (green lines). (*b*) When decreasing from high *υ* (blue lines), only relatively small increases to *β* maintains the *R*_0_ threshold, compared to decreases from small values of *υ* (green lines).

Figure 6 demonstrates how the nonlinear relationship between *R*_0_, *β* and *υ* enables the epidemic threshold to be exceeded for relatively small changes to parameter values. For example, for incremental decreases from high values of *β*, only relatively small increases to *υ* were required to maintain the system above the epidemic threshold (Fig. 6*a*). The converse was also true; incremental decreases from high values of *υ* only requiring a small increase in *β* to maintain the epidemic threshold (Fig. 6*b*).

## *R*_0_ thresholds: compartmentalization

**Fig. 7.**
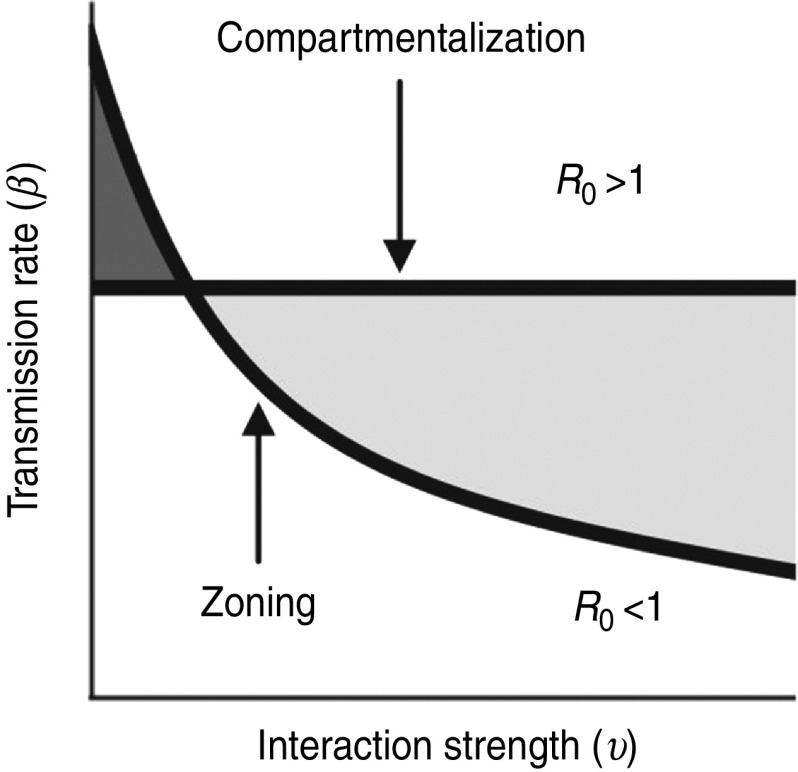
Schematic representation of the relative risk of infection spillover under compartmentalization. Dark-shaded area represents the additional risk of an outbreak (where *R*_0_>1) under compartmentalization and light-shaded area represents the additional risk of an outbreak (where *R*_0_>1) under zoning. The relative risk under compartmentalization can be increased through two ways: (i) a rightwards shift to the curve under zoning or (ii) a downwards shift to the line under compartmentalization.

Compartmentalization and zoning produce different characteristics for the epidemic threshold, as shown schematically in Figure 7. The different areas under the curves represent the difference in risk; the additional risk due to compartmentalization is shown by the dark-shaded region, while the additional risk from zoning is shown by the light-shaded region.

**Fig. 8.**
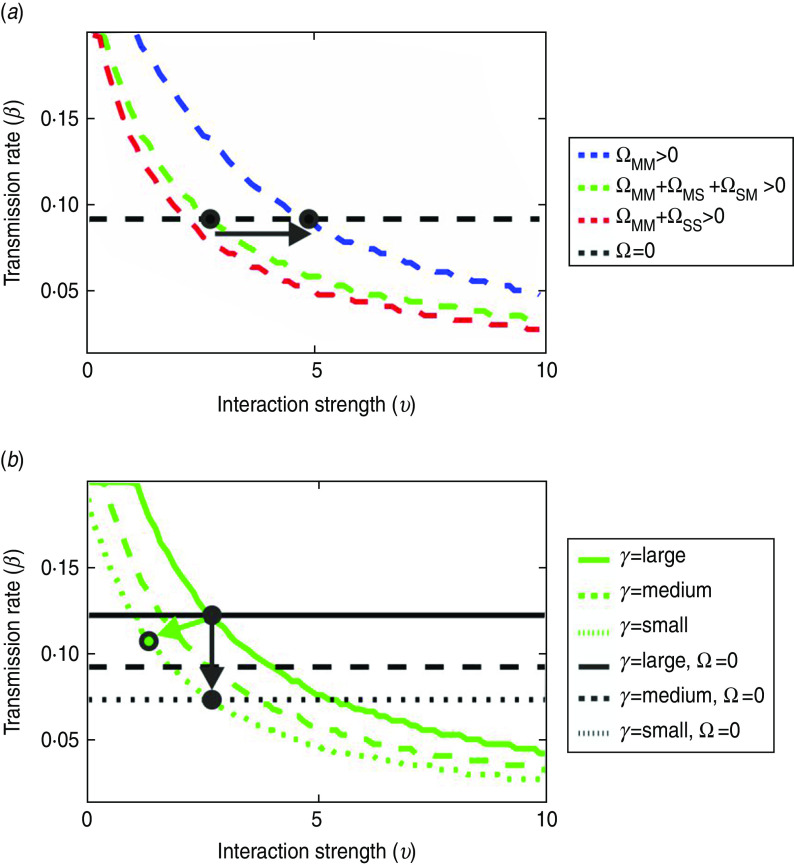
*R*_0_ thresholds under zoning (coloured curves) *vs.* compartmentalization (black lines). Ω_MM =_ links between multi-site premises, Ω_MS_ and Ω_SM_ = links between multi-site and single-site premises; Ω_SS_ = links between single-site premises. (*a*) For a medium infectious period corresponding to a premises depopulation rate of 0·25 (day^−1^), relative risk under compartmentalization was increased when the links enabling between-population interaction were reduced (from red to green to blue), as indicated by the arrow. Link types not shown in the legend are switched-off (i.e. Ω = 0). (*b*) For between-population interactions involving multi-site and single-site premises (i.e. Ω_MS_, Ω_SM_, Ω_SS_), the relative risk increased with the infectious period (from solid to dotted lines) under compartmentalization (black arrow) and zoning (green arrow). This model scenario assumed density-dependent spatial transmission occurred within a distance radius of 1 km.

Figure 8 overlays the *R*_0_ thresholds corresponding to compartmentalization (black horizontal lines) and zoning (coloured curves) to identify the parameter space where additional risk could be attributed to compartmentalization (see Fig. 7). Note that there is no sensitivity to *υ* under compartmentalization as between-population transmission is inhibited. A relatively greater risk under compartmentalization was found when: (*a*) removing the links enabling between-population interactions (i.e. decreasing the risk under zoning), or (*b*) increasing the infectious period. The impact of link types was greater than the infectious period, as shown by comparing the relative difference in arrow lengths in Figure 8*a* (for changes to the link types, Ω) and Figure 8*b* (for changes to the farm-level infectious period, 1/*γ*).

Although a greater risk for compartmentalization was found when fewer link types enabled between-population spread under zoning, the converse is also true; should zoning control be ineffective in inhibiting the risk of spread between populations through network links (i.e. because these connect many premises outside defined zones), compartmentalization would be a relatively more effective measure for protecting against an outbreak.

## DISCUSSION

Compartmentalization is a concept which could have major implications for poultry industries. However, there are no data to support its application during an outbreak, and so the refinement of its definition and exploration of its potential efficacy warrants further attention. In particular, the definition of epidemiological links and minimum distances to neighbouring farms could be made more specific through quantification of risk based on empirical data informing the structure of a specific poultry industry.

Our analysis centred on the fundamental structure underpinning poultry production in GB, as represented by a metapopulation of linked poultry company compartments and the SS premises within proximity. We used this modelling framework to assess the potential for an outbreak involving a compartment by quantifying the basic reproduction number, *R*_0_. While demographic stochasticity can be important in small populations, here we aimed to compare the *relative* potential for an outbreak under compartmentalization and zoning control; it was not our intention to assess the absolute risk of an outbreak occurring under one of these measures alone.

Ideally, these analyses should differentiate the different outbreak risks expected across poultry industry sectors (particularly layer hens and broiler chickens; Nick Sparks, personal communication). Indeed, previous analyses have highlighted the role of layer producers in the high farm-level network connectivity in GB through slaughterhouses and catching companies [11]. With respect to the poultry company-level network described here, the mean number of possible associated companies was similar between companies involved with layer and broiler production (29 and 26 company associations, respectively), although we note that layers had a greater minimum number (range 24–36 for layers *vs.* 2–36 for broilers).

With respect to farm-level network links, we focused on those between premises through catching teams and slaughterhouses as they are recognized to be of high risk for disease transmission within the GB poultry industry (during flock-thinning) and so our analyses largely reflect the commercial broiler chicken sector [9]. No data were available for other potential links between premises such as through feed delivery or egg collections in relation to layer hens. These additional links could also present epidemiological risks and could be added to future analyses concerning compartmentalization should national-level data describing these movements become available.

Regardless of production type, integrated poultry companies may be considered of low risk of disseminating infection through fomite-mediated mechanisms to external premises. However, we have identified links between different companies through shared slaughterhouse and catching companies at the farm level. This indicates the potential for disease transmission from these companies should compartmentalization be defined on the basis of shared ownership. By comparing the sensitivity of *R*_0_ across different farm-level factors, we found that outbreak risk was most sensitive to the type of links between populations (Fig. 5). The relatively greater probability of network links from a poultry company to external single sites (Fig. 3, Table 2) highlights the importance of considering these external premises when assessing the risk of disease spread from a compartment.

The ability to produce risk profiles for premises in this way is valuable when resources are restricted and their distribution must be prioritized. For example, *R*_0_ was relatively more sensitive to link-types (Ω) than to the farm-level infectious period (1/*γ*). This suggests that measures to restrict link types, representing industry-related movements, may have a greater impact on outbreak potential compared to the speed of outbreak detection. However, as restricting slaughterhouse or catching-team clientele to reduce between-farm connectivity may not be feasible, a more useful application might be to direct resources to forwards and backwards contact tracing during an outbreak.

When considering the potential effect of targeting control measures at network links, the nonlinear relationship between transmission rate (*β*) and interaction strength (*υ*) highlights how outbreak risk might be maintained even when the transmission rate is reduced (Fig. 6). Control measures that influence one aspect of transmission risk could alter another should farmers' respond to control measures with compensatory behaviour. These results imply the long-term impact could require further investigation if movement restrictions (which could alter *β, υ* or both) were to be a condition for compartmentalization.

Poultry companies typically locate their premises out of close proximity to one another, or to other non-company premises, to meet biosecurity requirements (Nick Sparks, personal communication); however, quantitative evidence supporting the minimum inter-farm distance of 400 m in the GB guidelines [26] is lacking. Our results show that even if all network links were to be inhibited under compartmentalization, the risk of an outbreak may be maintained via spatially mediated airborne spread alone (Fig. 8). Outbreak conditions were found where the risk was relatively greater under compartmentalization than under zoning control (where instead spatial links were inhibited). We could not have predicted that the risk under zoning control would decrease as the risk under compartmentalization increased for our combination of parameter ranges.

Although most between-farm distances were large (>10 km), and *R*_0_ was relatively insensitive to spatial separation of farms, the sensitivity of *R*_0_ was heightened under density-dependence and resulted in the potential for relatively greater risk through compartmentalization compared to zoning control. This relatively greater risk under compartmentalization was found for airborne transmission within a distance of 1 km – only a short increase above the current recommended minimum distance of 400 m for compartments in GB [28]. At this relatively short distance, multi-site farms were more likely to be in the proximity of other multi-site farms external to their company. With greater knowledge of the likely transmissibility of HPAI in GB, as well as farm-level biosecurity practices, our analysis could provide a framework to inform evidence-based criteria for compartments in GB. Furthermore, this framework could be used as a decision-making tool in the event of an outbreak to inform whether compartmentalization is preferable to zoning as a control measure against HPAI.

## CONCLUSIONS

The current requirements enabling farms to achieve compartment status are not specific to a country's poultry industry structure, or to the likely mechanism enabling epidemiological links between farms. Our analyses highlight the potential for infection spread from compartments via: (i) epidemiologically relevant network links between premises of unrelated companies and (ii) through airborne spread to single-site premises in proximity. With greater knowledge of the likely transmissibility of HPAI in GB, these analyses could help define an appropriate evidence-based minimum between-farm distance to inform more specific guidelines for compartmentalization in GB.

## Supplementary Material

Supplementary Material Supplementary information supplied by authors.

## Supplementary material

For supplementary material accompanying this paper visit https://doi.org/10.1017/S0950268813002963. click here to view supplementary material
