# Supplementary material for: A metapopulation model for highly pathogenic avian influenza: implications for compartmentalization as a control measure
Source: Epidemiol Infect. 2013 Dec 5;142(9):1813–25. doi: 10.1017/S0950268813002963 (PMC4102102; doi:10.1017/S0950268813002963)
Supplement: Supplementary Material — Supplementary information supplied by authors. [file S0950268813002963sup001.doc]

**SUPPLEMENTARY MATERIAL**

**Appendix A. Scaling link-type probabilities**

The Poultry Network Database (PND) was used to compute the frequency at which each possible link-type (across multi-site and single-site premises) occurred across the GB poultry population. However, this database is not representative of the entire British poultry industry [12]. Therefore the frequency of each link-type was re-scaled to that expected according to the distribution of multi-site (*j*=M) and single-site (*j*=S) premises recorded by the more demographically representative Great Britain Poultry Register (GBPR) [24]; this database held 24,119 premises records (after excluding 4075 possible duplicate entries) in the 2009 extract available for this study.

The link-type probabilities informed by the PND (described in section 2.4) were multiplied by the known frequency of multi-site and single-site premises recorded by the GBPR, generating a more representative frequency of link types. The sampling of individual poultry premises from the population was then carried out as a binomial process (using the *binofit* function in Matlab® v.7.8.0, The MathWorks Inc., Natwick, MA, USA), representing the sampling of links per multi-site and single-site premises through a set of independent trials.

This process generated maximum likelihood estimates for the probability of sampling the expected frequency of (scaled) links in 24,000 sampling trials (to match the population size informed by the GBPR) for each link-type; the upper limit of the corresponding 95% confidence intervals informed the link-type probabilities in Table 2 of the main text. We chose the upper limit in order to represent a worst case scenario in terms of the risk of infection transmission. We use these scaled link-type probabilities to inform a weighting applied to the transmission rate parameter in the S-I-R model.

**Appendix B. Computing the basic reproduction number, *R*_0_**

The basic reproduction number (*R*_0_) was computed as the dominant eigenvalue of the Next Generation matrix for the ODE system described in section 2.5 of the main text as follows:

$$R_{0}=p\left(\begin{matrix} \frac{\beta'\sigma_{1,MS}+\beta\omega_{1,MS}}{\gamma} & \frac{\beta'\sigma_{1,SS}+\beta\omega_{1,SS}}{\gamma} & \frac{\beta\Omega_{1,MS}\upsilon}{\gamma} & \frac{\beta\Omega_{1,SS}\upsilon}{\gamma} \\ \frac{\beta'\sigma_{2,MS}+\beta\omega_{2,MS}}{\gamma} & \frac{\beta'\sigma_{2,SS}+\beta\omega_{2,SS}}{\gamma} & \frac{\beta\Omega_{2,MS}\upsilon}{\gamma} & \frac{\beta\Omega_{2,SS}\upsilon}{\gamma} \\ \frac{\beta\Omega_{1,MS}\upsilon}{\gamma} & \frac{\beta\Omega_{1,SS}\upsilon}{\gamma} & \frac{\beta'\sigma_{1,MS}+\beta\omega_{1,MS}}{\gamma} & \frac{\beta'\sigma_{1,SS}+\beta\omega_{1,SS}}{\gamma} \\ \frac{\beta\Omega_{2,MS}\upsilon}{\gamma} & \frac{\beta\Omega_{2,SS}\upsilon}{\gamma} & \frac{\beta'\sigma_{2,MS}+\beta\omega_{2,MS}}{\gamma} & \frac{\beta'\sigma_{2,SS}+\beta\omega_{2,SS}}{\gamma} \end{matrix}\right)$$

where MS refers to multi-site premises and SS refers to single-site premises. Full details of parameter values and definitions are provided in Table 1 of the main text.
